# When group grievances become personal: The neural correlates of group and personal rejection

**DOI:** 10.3758/s13415-024-01257-x

**Published:** 2025-01-07

**Authors:** Luis Marcos-Vidal, Helena Gil-Buitrago, Irene Cisma, Rosamunde C. Hendricks, Scott Atran, Clara Pretus

**Affiliations:** 1 Hospital del Mar Research Institute, 08003 Barcelona, Spain; 2 Department of Psychobiology and Methodology of Health Sciences, Universitat Autònoma de Barcelona, Carrer de La Fortuna, 08193 Barcelona, Spain; 3 Changing Character of War Centre, University of Oxford, Oxford, UK; 4 Center of Conflict Studies and Field Research, ARTIS International, St. Michaels, MD USA

**Keywords:** Social exclusion, Rejection, Group discrimination, Ostracism, FMRI, Social neuroscience

## Abstract

Witnessing rejection against one’s group can have similar impacts on psychological distress and aggression as experiencing rejection personally. In this study, we investigated the neural activity patterns of group rejection and whether they resemble those of personal-level rejection. We first identified the neural correlates of social rejection (exclusion based on negative attention) compared with ostracism (exclusion based on lack of social connection) and then compared group-level to personal-level rejection. We employed a novel social exclusion task, “RateME,” to induce group and personal rejection and Cyberball to evoke ostracism during fMRI scans. Our results showed that personal rejection increased activity in regions associated with autobiographical memory and self-identity, such as the dorsomedial prefrontal cortex and the lingual gyrus, whereas ostracism engaged areas related to social pain and salience, including the anterior cingulate cortex and the insula. Additionally, group and personal-level rejection elicited similar neural activity patterns, regardless of participants’ fusion with the rejected groups. Therefore, group membership seems sufficient for group rejection to trigger self-referential processing pathways similar to those activated by personal rejection. Our findings highlight the overlapping neural mechanisms underlying personal and group-level grievances, which may explain the detrimental effects of group rejection on aggression, extremism, and intergroup conflict.

**Supplementary Information:**

The online version contains supplementary material available at 10.3758/s13415-024-01257-x.

## Introduction

Group discrimination, or creating, maintaining, or reinforcing an advantage for certain groups and their members over others (Dovidio & Ikizer, 2019), can lead to higher willingness to engage in extreme pro-group action (Gómez et al., 2011; Pretus et al., 2018). Interviews with prospective suicide bombers and their supporters reveal that violent extremism often flourishes among those who feel humiliated in their own lives or identify with the humiliation of others (Atran & Stern, 2005). Group discrimination also contributes to enduring intractable conflict. For example, perceived discrimination in the form of political sanctions led to increased support for Iran’s nuclear program (Dehghani et al., 2010), whereas Palestinians who felt more humiliated by peace deals were less likely to support them (Ginges & Atran, 2008). Besides, witnessing one’s group being rejected, a short-term form of discrimination, has been found to have similar but experimentally dissociable effects on psychological distress and hostility compared with directly experiencing rejection oneself (Marcos-Vidal et al., 2024a, 2024b). However, the effects of group rejection on brain activity, and whether they mirror those exerted by personal rejection, have not been studied yet. The current research aims to explore the neural response to group-level as compared to personal-level rejection.

Social rejection, along with ostracism, are different forms of social exclusion. Social rejection is the act of dismissing someone by using negative attention or explicit declarations of dislike (Wesselmann & Williams, 2017; Williams, 2007), whereas ostracism involves ignoring or depriving individuals of attention and social connection (Wesselmann & Williams, 2017). Most research on social exclusion has focused on ostracism using Cyberball, a virtual ball-tossing game where participants play with other virtual players (Williams et al., 2000). Thus, despite its significance, the literature on social rejection is relatively limited, and no standardized task exists to measure it (Moor et al., 2010; Woo et al., 2014).

Relatedly, most of the studies examining the neural correlates of social exclusion have focused on ostracism. Initial studies on ostracism found neural activation in areas related to pain and disgust, such as the dorsal Anterior Cingulate Cortex (dACC) and the Anterior Insula (Rainville et al., 1997; Eisenberger et al., 2003; Onoda et al., 2010; Eisenberger, 2012). However, recent research has questioned the role of the dACC in ostracism, suggesting instead a more prominent role of the ventral Anterior Cingulate (vACC) and superior and inferior frontal gyri together with the Anterior Insula (Mwilambwe-Tshilobo & Spreng, 2021; Sebastian et al., 2011). These regions have been associated with cognitive and affective responses to social exclusion (Grecucci et al., 2013; Rotge et al., 2015), suggesting an essential role of emotional regulation processes following ostracism (DeWall et al., 2011). Another study found greater activity in the dorsomedial and dorsolateral prefrontal cortex, as well as the left temporoparietal junction, in response to witnessing ostracism directed at outgroup (versus ingroup) targets using Cyberball (Lasko et al., 2022). These neural pathways are related to social exclusion, but their generalizability from ostracism to other forms of exclusion remains unclear.

Only a few studies have examined the neural effects of personal rejection. One of them induced personal rejection by showing participants images of their ex-partners, finding increased activity in areas related to self-referential processing, such as the Dorsomedial Prefrontal Cortex (DMPFC), the right Temporoparietal Junction (TPJ), and the Precuneus (Woo et al., 2014). Additionally, they found greater activity in areas involved in negative emotion regulation, including the thalamus, inferior frontal gyrus, and supplementary motor area (SMA) (Woo et al., 2014). In contrast, other studies did not find any significant neural activity related to social rejection using the “Chatroom Task,” a feedback task where participants received positive or negative feedback by a series of strangers (Moor et al., 2010; Guyer et al., 2012). The authors suggest that the task may have evoked stronger responses if the feedback had been linked to tangible aspects of the participants’ personality or if it had come from peers rather than strangers. Overall, different types of social exclusion may be driven by distinct neural processes, which need to be identified.

To our knowledge, no studies have evaluated how the brain responds to group rejection. Several brain regions are thought to play an important role in processing social information associated with group membership, including three subregions of the medial prefrontal cortex (MPFC): the ventromedial prefrontal cortex (VMPFC), the DMPFC, and portions of the anterior cingulate cortex (ACC) (Amodio & Frith, 2006; Cikara et al., 2014; Molenberghs & Morrison, 2014). Greater activity in the VMPFC and the DMPFC has been related to ingroup favoritism, a relationship that was stronger in participants who felt a visceral connection with the ingroup (Apps et al., 2018), also known as identity fusion (Swann et al., 2009). However, further research is needed to better understand how the neural correlates of group rejection may vary as a function of how much people identify with the rejected group.

### Current research

The present study aims to assess the neural correlates of group rejection. For that, we experimentally induce feelings of group rejection using a novel social evaluation task called “RateME” (Marcos-Vidal et al., 2024a, 2024b). “RateME” includes two settings, “Group RateME” and “Personal RateME,” designed to elicit feelings of group and personal-level rejection, respectively. To characterize the brain response to group rejection, we will first identify its neural correlates and compare them to personal rejection and ostracism. We elicit feelings of personal rejection with “Personal RateME” and ostracism with the well-established Cyberball game (Williams & Jarvis, 2006). Next, we distinguish between group and personal-level rejection by examining the brain response to “Group RateME” and “Personal RateME.” As a secondary goal, we assess whether neural responses to group rejection mirror those elicited by personal rejection among individuals fused with rejected groups. Finally, we explore potential correlations between the neural response to group rejection and intergroup attitudes, such as perceived outgroup threat and radicalism.

We hypothesize that social rejection will be marked by a specific pattern of neural activation different from that associated with ostracism. Moreover, we expect neural responses to group rejection to mirror those elicited by personal rejection among individuals with fused identities. Finally, we hypothesize that participants with more exclusionary intergroup attitudes, such as higher radicalism and perceived outgroup threat, will be more responsive to group rejection, especially in terms of neural activity in areas associated with identity processes. The findings of this study will help to clarify whether group and personal grievances are processed in similar ways. This will contribute to addressing the question of whether group rejection should be regarded as a risk factor for mental health and violent extremism.

## Methods

The study materials and code employed in the analyses are available at https://osf.io/pc4fs/. A standalone application of the RateME task is available upon request. The dataset is publicly available at https://openneuro.org/datasets/ds005375. The hypotheses and analysis plan of this study were not preregistered.

This research was approved by the Ethics Committee on Human and Animal Experimentation at the Universitat Autònoma de Barcelona according to the Declaration of Helsinki guidelines (Ref. 6530).

### Participants

We recruited a sample of 58 adults residing in Spain (29 women) through social media and local advertisements. Participants were aged 18 to 55 years old (*M* = 32.01, *SD* = 11.22). Half of the sample reported feeling fused with Spain (N = 28). Fused and nonfused participants were matched for age (t(56) = 0.48, *p* = 0.635) and gender (χ^2^(1) = 0.07, *p* = 0.79) (Table 1). Additionally, to ensure participants’ suitability, we verified via phone screening that none of the individuals had any neurological or mental health disorders. Finally, an exclusion criterion was set for high in-scanner motion (framewise displacement greater than 0.5 mm), but no participants reached this threshold.

**Table 1:** Participants' demographics and descriptive statistics of movement during Cyberball, Personal, and Group RateME

Variable	Total sample (N / mean ± SD)	Fused (N / mean ± SD)	Nonfused (N / mean ± SD)	t/${\varvec{\chi}}$(***P***-value)
**Gender**	58	28	30	0.07 (0.793)
Male	29	13	16	
Female	29	15	14	
**Age (mean ± SD)**	32.02 ± 11.22	32.75 ± 11.3	31.33 ± 11.29	0.48 (0.635)
Male	33.03 ± 11.35	32.85 ± 12.08	33.19 ± 11.12	
Female	30.94 ± 11.19	32.67 ± 11	29.21 ± 11.51	
**Education (mean ± SD)**	5.47 ± 1.19	5.69 ± 1.26	5.22 ± 1.09	1.42 (0.163)
Male	5.5 ± 0.99	5.83 ± 1.11	5.21 ± 0.8	
Female	5.43 ± 1.41	5.57 ± 1.4	5.22 ± 1.48	
**FD Cyberball**	0.14 ± 0.05	0.14 ± 0.04	0.14 ± 0.05	− 0.24 (0.811)
**FD Personal RateME**	0.15 ± 0.05	0.16 ± 0.06	0.14 ± 0.05	0.94 (0.353)
**FD Group RateME**	0.15 ± 0.07	0.14 ± 0.05	0.16 ± 0.08	− 0.72 (0.473)

### Protocol

All participants completed an initial survey on demographic information and group identification measures. Next, participants were asked to complete a pre-scan survey in preparation for the RateME tasks. Details of this questionnaire and cover story can be found in Supplementary Materials. Then, participants were invited to the fMRI facilities where they completed the three experimental tasks described below (Cyberball, Personal RateME, and Group RateME) in separate runs in a randomized order. After the experimental task, participants completed an online questionnaire measuring intergroup attitudes.

### Behavioral measures

The following measures were obtained before the fMRI task. Average ratings for each measure can be found in Table 2.

**Table 2:** Average ratings of the behavioral measures

Scale	Fused (mean / SD)	Nonfused (mean / SD)	Total (mean /SD)	t(*p*-value)
ARIS activism	4.11 / 1.07	3.21 / 1.51	3.64 / 1.38	2.66 (0.01)
ARIS radicalism	2.83 / 1.31	3.54 / 1.28	3.20 / 1.33	− 2.12 (0.038)
Outgroup threat Perception	2.12 / 1.05	2.39 / 0.88	2.26 / 0.97	− 1.08 (0.285)
Collective Narcissism	3.19 / 1.03	2.75 / 1.04	2.96 / 1.05	1.65 (0.105)
Spain Physical Formidability	3.25 / 1.14	3.00 / 1.08	3.12 / 1.11	0.87 (0.387)
Spain Spiritual Formidability	3.75 / 1.27	2.73 / 1.28	3.22 / 1.36	3.09 (< 0.01)
EU Spiritual Formidability	3.61 / 1.40	3.20 / 1.27	3.40 / 1.34	1.19 (0.239)
EU Physical Formidability	4.79 / 1.10	4.07 / 1.26	4.41 / 1.23	2.36 (0.022)
Costly sacrifice	2.61 / 2.73	2.07 / 2.48	2.337 / 2.59	0.8 (0.426)

#### Identity fusion

Identity fusion was assessed in relation to Spain and with the European Union prior to the fMRI task using the Swann et al. (2009) pictorial measure. This measure includes five pairs of circles with different degrees of overlap, which represent the relationship between the participant (small circle) and the group (big circle). Respondents were asked to convey which pair of circles best represents their relationship with Spain and the EU. The scale was recoded into a binary variable with 1 for those participants with maximum identity fusion and 0 otherwise, in line with Swann et al. (2009). Individuals fused with a group have been found to display qualitatively different behaviors compared with nonfused individuals, such as willingness to engage in extreme behaviors on behalf of the group, especially after undergoing exclusion (Gómez et al., 2011). Thus, we adopted a binary approach to assess potential neural activity differences between fused and non-fused individuals during the fMRI task. The following intergroup attitude measures were obtained after the fMRI task:

#### Perceived threat by the EU 

We measured perceived threat by the EU with an adapted version of the Outgroup Threat Perception Scale (OTPS) (Luque et al., 2012), which includes a symbolic threat subscale (e.g.*, Educational values such as values taught in schools are under threat because of the EU*) and a realistic threat subscale (e.g., *Access to the public aid system, such as housing aid and unemployment aid, is under threat because of the EU*). Participants indicated how much each of these dimensions was in danger from 1 = Not in danger at all to 5 = Completely in danger with 6 items.

#### Collective narcissism 

We obtained a measure of collective narcissism with an adapted version of the Collective Narcissism Scale (CNS) (de Zavala et al., 2009). Participants indicated their agreement from 1 = Strongly Disagree to 6 = Strongly Agree with 5 items (e.g., *Spain deserves special treatment*; *It really makes me angry when others criticize Spain*).

#### Activism and radicalism 

We adapted the Activism and Radicalism Intention scale (ARIS) (Moskalenko & McCauley, 2009) to the context of Spain and the European Union. Participants indicated their agreement from 1 = Strongly Disagree to 7 = Strongly Agree with 6 items assessing support for nonviolent and violent intergroup action, including *I would join an organization that fights for Spain's intergroup and legal rights with regards to the European Union*, and *I would continue to support an organization that fights Spain's political and legal rights with regards to the European Union even if the organization sometimes resorts to violence*. Following Moskalenko and McCauley (2009), the first three items were averaged into an activism composite score (Cronbach’s alpha = 0.88), and the last three items were averaged into a radicalism composite score (Cronbach’s alpha = 0.73).

#### Costly sacrifices 

We measured the number of costly sacrifices that participants were willing to engage in to defend the UK with an adapted version of the Costly Sacrifices Scale ​(Atran et al., 2014). Participants indicated the sacrifices they were willing to engage in among 7 items: “Lose my job or source of income”; “Use violence”; “Go to jail”; “Let my children suffer physical punishment”; “Die”; “None of the above”; and “Prefer not to say.” Responses were recoded into a binary variable as 1 if one (or more) of the proposed actions was chosen and 0 if”None of the above” or”Prefer not to say” were chosen.

#### Spiritual and physical formidability 

We adapted the Spiritual and Physical Formidability Scale (Tossell et al., 2022) to the context of Spain and the European Union. Participants indicated their perception of the physical and spiritual formidability of both Spain and the European Union by using a visual scale that depicts a series of male bodies that vary from relatively small and weak to large and strong (1 = Low formidability to 6 = More formidability).

#### fMRI task

##### Group RateME

**Fig. 1:**
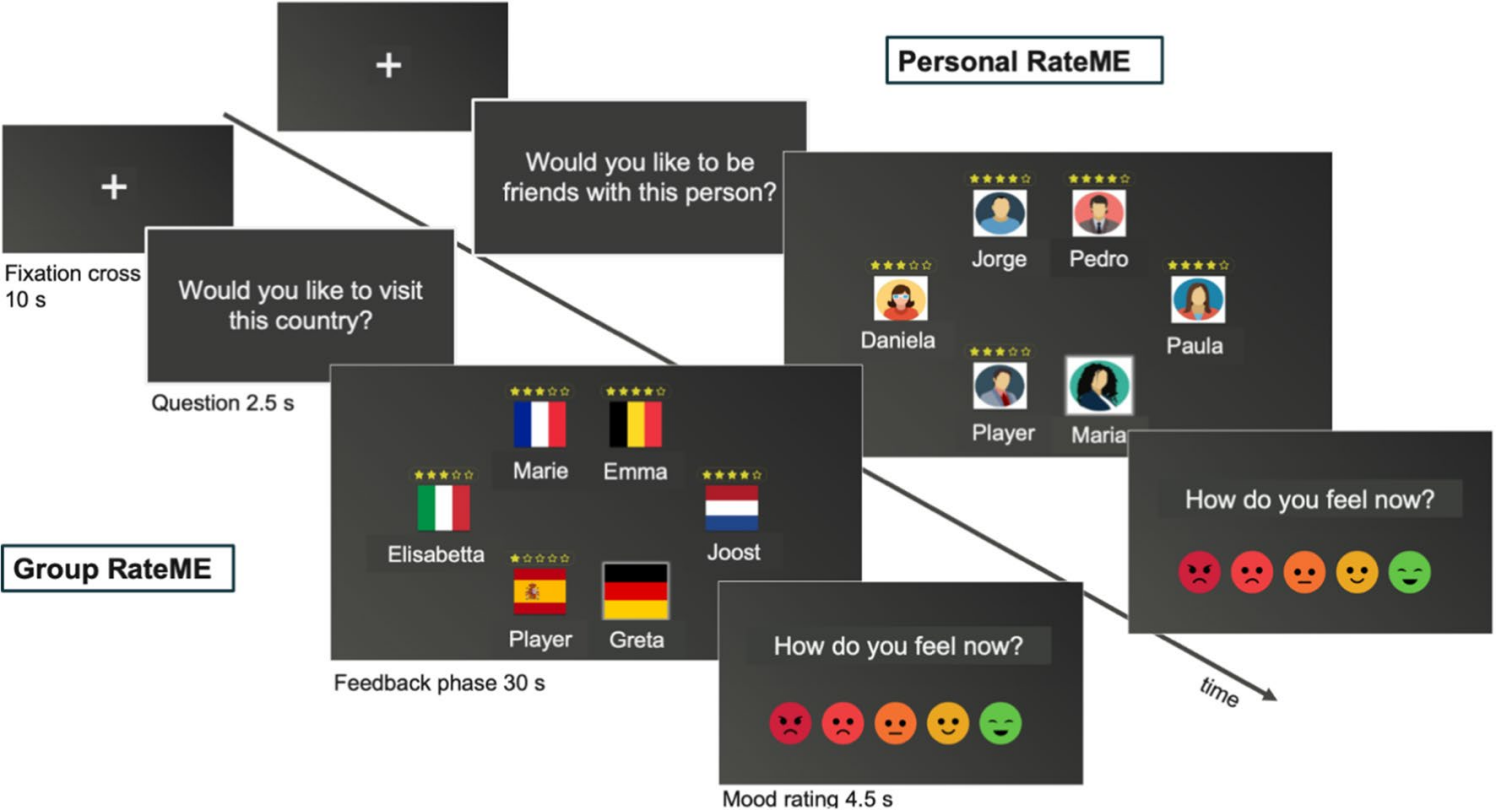
Overview of the Personal RateMe (**A**) and Group RateME paradigm (**B**)

In the Group RateME task, participants were invited to participate in a group setting with other players represented by a flag and a traditional name from the same country (e.g., Joost for Netherlands or Greta for Germany). Participants were represented by the Spanish flag, and the other avatars by flags of other European countries (Fig. 1). Participants visualized each of the questions they had answered in the pre-scan survey (Table 3) followed by the other players’ ratings on their country (Spain) compared with the other players’ countries (Fig. 1; Table 3). In the exclusion condition, other European countries received positive ratings on all dimensions, while Spain received various degrees of negative ratings from all other players. In the inclusion condition, Spain received similarly positive ratings as all other countries. The duration of this game was around 7 min.

**Table 3:** Rating items used in RateME (Translated from Spanish)

Task	Rating criteria
Group RateME	Would you visit this country?
	Would you live in this country for a while?
	Are people in this country efficient?
	Are people in this country interesting?
	Are people in this country fun?
	Are people in this country kind?
Personal RateME	Would you spend time with this person?
	Would you be friends with this person?
	Is this person intelligent?
	Is this person interesting?
	Is this person fun?
	Is this person kind?

##### Personal RateME 

In the Personal RateME task, participants were invited to participate in a group setting with five other avatars (Fig. 1). Each avatar was represented by a name (including the participant's name), and a colored silhouette. Participants visualized each of the questions they had answered in the prescan survey (Table 3) followed by the other players’ ratings on their profile compared with the other players’ profiles (Fig. 1; Table 3). In the exclusion condition, participants received negative feedback on their profile, while the other avatars received various degrees of positive feedback. In the inclusion condition, participants received comparable levels of positive feedback as the other players. The mean duration of the game was 7 min.

##### Cyberball 

Cyberball is a ball-tossing game where participants play with another three avatars. The avatars had traditional Spanish names positioned next to a colored silhouette. Participants in the exclusion condition did not receive the ball and were thus ignored, while they received the ball approximately one-third of the time in the inclusion condition. We used a custom version of Cyberball specifically designed to be synchronized with the fMRI scanner.

Participants completed the three experimental tasks (Group RateME, Personal RateME, and Cyberball) in separate runs and were subjected to both exclusion and inclusion conditions in each task. Each run consisted of ten 30-s blocks, including five exclusion blocks and five inclusion blocks presented in a randomized order. Before the RateME blocks, participants were presented with the question that was going to be rated for 2.5 s. Likewise, after each block, we assessed participants’ mood using a 5-point scale with different colored smiley faces, which was presented for 4.5 s. This mood question was separated from the subsequent block by a 10-s interblock interval displaying a fixation cross (Fig. 1).

#### fMRI acquisition & preprocessing

A detailed description of the acquisition parameters together with the preprocessing steps as provided by the boilerplate can be found in Supplementary Material. Briefly, fMRI data were preprocessed by using fMRIPrep version 20.2.0 (Esteban et al., 2018), a Nipype-based tool (Gorgolewski et al., 2011). This pipeline includes bias-field correction, coregistration, segmentation, motion-correction, and spatial normalization.

#### fMRI GLM analysis

Both individual- and group-level analyses were performed by using AFNI (version 24.0.12; Cox, 1996; Cox & Hyde, 1997). First, fMRI data were smoothed with a Gaussian kernel of 6-mm full-width at half maximum. Then, we performed the individual level analysis with a linear model including boxcar regressors for the inclusion and exclusion blocks separately, and additional regressors for the mood question blocks, 12 motion parameters and mean white matter, cerebrospinal fluid, whole brain signal, and a fifth-order polynomial to remove very low-frequency drift. In the Cyberball task, we also modeled participants’ motor response with a regressor tracking when participants had pressed a button to toss the ball. From these analyses, we obtained beta maps of exclusion, inclusion, the exclusion versus inclusion contrast, and a t map of exclusion versus inclusion for each participant and each different task.

We then performed group-level analyses for each task separately by using 3dMEMA (Chen et al., 2012). The linear models included age, gender, and mean framewise displacement as covariates of no interest, and in the Group RateME models, we also included identity fusion to explore whether it modulated exclusion effects. To compare exclusion effects across different tasks, we fitted two different linear models: one that compared exclusion *versus* inclusion in Personal RateME against exclusion *versus* inclusion in Cyberball to assess the difference between personal rejection and ostracism, and another model that compared exclusion *versus* inclusion in Personal RateME against exclusion *versus* inclusion in Group RateME to examine differences between personal and group-level rejection. All models included age, gender, and mean framewise displacement as covariates of no interest. The second model also included identity fusion.

Each voxel-wise analysis was corrected by multiple comparisons at the cluster level with a Family-wise Error Rate of 0.05 (p_FWE < 0.05). For that, we used 3dClustSim and considered as significant those clusters of at least 24 voxels with *p* < 0.001.

#### Correlations with intergroup attitudes

Finally, we assessed whether intergroup attitudes were associated with the neural responses to group exclusion. We restricted these analyses to the Regions of Interest (ROIs) where identity fusion modulated the level of neural activity during exclusion. We did so to examine whether identity fusion mediated the relationship between these regions and intergroup attitudes. However, we only found direct (nonmediated) effects between some of these ROIs and intergroup attitudes. We describe these analyses below.

To select the ROIs, we thresholded the statistical map of the effect of identity fusion in the group rejection GLM model described in the previous section with p < 0.005 and a cluster extent of 24 voxels. We then selected the ROIs from the meta-analytic functional co-activation parcellation (https://neurovault.org/images/395092/) that overlapped with the thresholded map. This functional parcellation was created based on meta-analytic functional co-activation of the neurosynth database36 (Yarkoni et al., 2011) and is publicly available at neurovault (https://neurovault.org/images/395092/). After that, we averaged the exclusion *versus* inclusion beta values (of the group RateME task) in each of the ROIs for each participant, obtaining one value of group exclusion for each participant and each ROI.

We fitted one linear model per ROI and intergroup attitudes variable (activism, radicalism, outgroup physical threat, outgroup symbolic threat, collective narcissism, and physical and spiritual formidability of the ingroup). Each of these models included the ROI exclusion pattern as dependent variable and age, gender, framewise-displacement, identity fusion, and the intergroup attitude variable as independent variables. We then tested the effect of intergroup attitudes and corrected the results of these tests by using FDR correction (Benjamini & Hochberg, 1995) for each of the dependent variables (ROIs).

## Results

### Behavioral analysis

We first examined the effect of exclusion blocks on participants in the three different tasks. We found that participants reported more negative mood after exclusion blocks compared with inclusion in the three tasks (*b* =  − 1.750, *SE* = 0.144*, t*(*275*) =  − 12.139*, p* < 0.001). Additionally, the negative effect of exclusion on mood was lower in group RateME than Cyberball *(b* = 0.500, *SE* = 0.204*, t*(*275*) = 2–453*, p* = 0.015), and mood was overall more positive in group RateME than Cyberball (*b* = 0.420, *SE* = 0.144*, t*(*275*) = 2.911*, p* = 0.004).

#### Neural activity patterns of personal rejection compared with ostracism

**Fig. 2:**
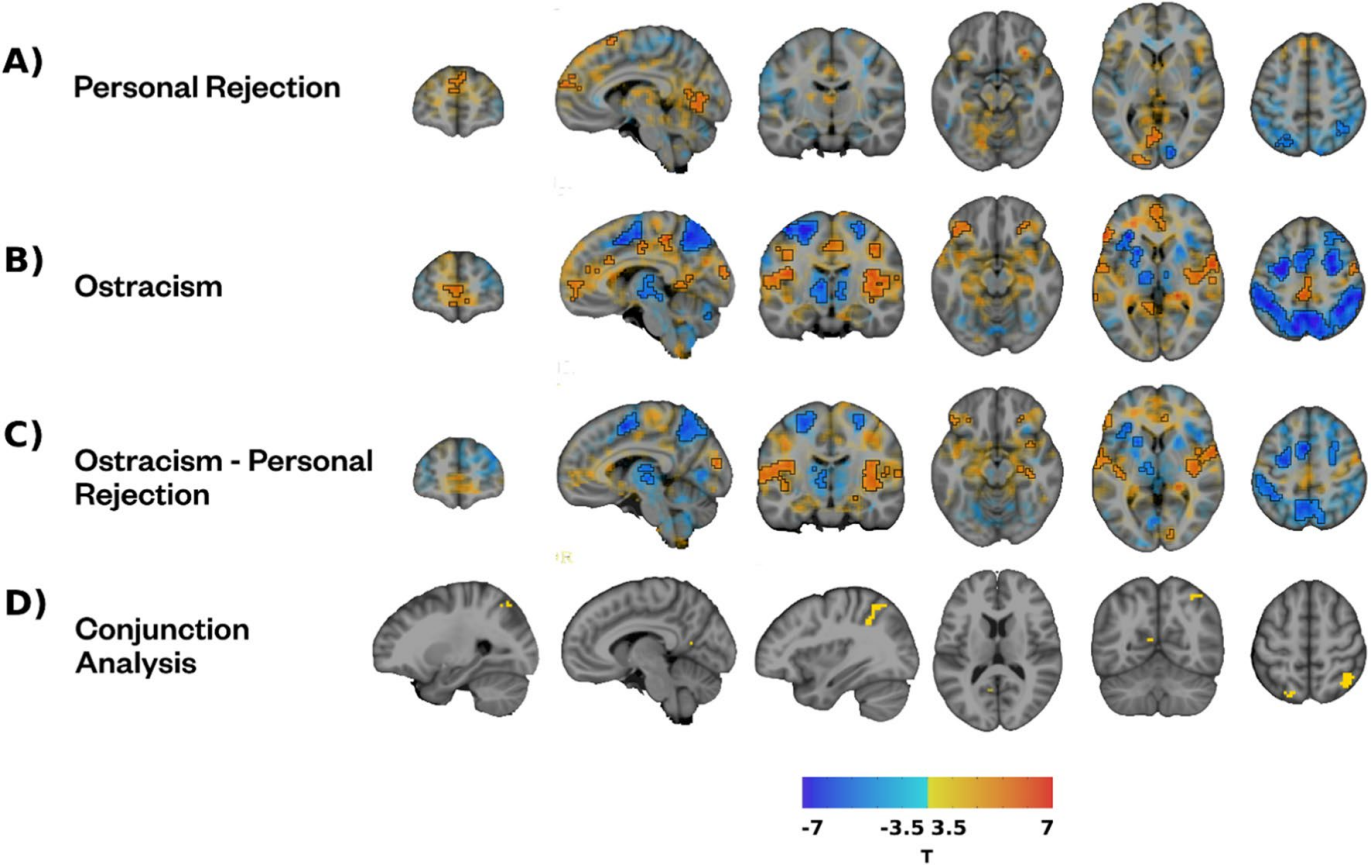
Activation maps characterizing personal exclusion. *Note.* **A**) Activity related to personal rejection as the difference between exclusion and inclusion in Personal RateME. **B**) Activity related to ostracism as the difference between exclusion and inclusion in Cyberball. **C**) Difference between ostracism and personal rejection. **D**) Results of the conjunction analysis between ostracism and personal rejection

Personal RateME elicited both increases and decreases in brain activity during exclusion compared to inclusion blocks. Specifically, we found greater neural activity in multiple clusters encompassing areas of the left occipital cortex, the Superior Frontal Gyrus (SFG), and the bilateral SMA during exclusion (Table 4; Fig. 2). We also found some areas that exhibited lower activation during exclusion compared with inclusion, including an area of the right Occipital Cortex, clusters in the Superior Parietal Cortex, and a region encompassing the left Precentral and Postcentral Gyri (Table 4; Fig. 2).

**Table 4:** Neuroimaging results of the contrast between exclusion and inclusion in the Personal RateME task

Cluster	Size	T mean	Overlap	ROI location
Exclusion > Inclusion				
1	147	4.11	44.10%	Left Lingual gyrus
			24.90%	Left Middle occipital gyrus
			20.00%	Left Calcarine fissure and surrounding cortex
2	39	3.96	83.00%	Left Superior frontal gyrus, medial
			11.30%	Right Superior frontal gyrus, medial
3	37	4.12	68.80%	Right Supplementary motor area
			31.20%	Left Supplementary motor area
Inclusion > Exclusion				
4	107	−4.06	36.10%	Right Cuneus
			33.00%	Right Calcarine fissure and surrounding cortex
5	60	−3.92	46.80%	Right Inferior parietal gyrus
			29.80%	Right Superior parietal gyrus
6	25	−3.8	96.10%	Left Superior parietal gyrus
7	25	−3.85	73.60%	Left Precentral gyrus
			26.40%	Left Postcentral gyrus

We found a different pattern of brain activity related to ostracism by comparing the exclusion and the inclusion conditions during Cyberball. Ostracism was associated with increased activity in the right Insula, temporal areas, the Cingulate Cortex, the ventromedial SFG, the Ventrolateral Prefrontal Cortex, the Orbitofrontal Cortex, left Calcarine fissure, and the left Precuneus (Table 4; Fig. 2). We also found reduced neural activity during exclusion compared with inclusion in parietal areas, such as the Inferior Parietal Cortex, bilateral Precuneus, bilateral Superior Parietal Cortex, as well as prefrontal areas, such as the lateral Superior Frontal Cortex, the bilateral Precentral Gyrus, and the SMA, and subcortical areas (Table 5; Supplementary Table 1; Fig. 2).

**Table 5:** Neuroimaging results of the contrast between exclusion and inclusion in Cyberball

Cluster	Size	T mean	Overlap	ROI location
Exclusion > Inclusion				
1	541	4.33	35.9%	Right Rolandic operculum
			27.6%	Right Superior temporal gyrus
			13.8%	Right insula
2	316	4.24	42.4%	Left Superior temporal gyrus
			29.1%	Left Rolandic operculum
			14.1%	Left Postcentral gyrus
3	163	3.95	30.4%	Left Anterior cingulate cortex, supracallosal
			15.3%	Left Superior frontal gyrus, medial
			15.3%	Left Anterior cingulate cortex, pregenual
4	159	4.33	31.1%	Left Inferior frontal gyrus, triangular part
			26.3%	Left Inferior frontal gyrus, orbital part
			11.0%	Left Lateral orbital gyrus
			10.0%	Left Temporal pole: superior temporal gyrus
5	154	4.07	45.4%	Left Middle cingulate & paracingulate gyri
			31.6%	Right Middle cingulate & paracingulate gyri
			11.0%	Right Supplementary motor area
Inclusion > Exclusion				
6	2135	− 4.73	17.9%	Left Inferior parietal gyrus
			14.4%	Left Precuneus
			13.3%	Right Inferior parietal gyrus
			11.3%	Right Precuneus
			10.6%	Left Superior parietal gyrus
7	697	− 4.63	50.0%	Right Superior frontal gyrus, medial
			23.6%	Right Middle frontal gyrus
			11.1%	Right Inferior frontal gyrus, opercular part
8	600	− 5.39	30.7%	Left Precentral gyrus
			26.3%	Left Superior frontal gyrus, medial
			24.6%	Left Supplementary motor area
9	166	− 4.32	62.3%	Left Precentral gyrus
			13.1%	Left Inferior frontal gyrus, triangular part
			12.2%	Left Middle frontal gyrus
			11.5%	Left Inferior frontal gyrus, opercular part
10	137	− 4.32	43.2%	Left Insula
			41.9%	Left Lenticular nucleus, Putamen

Comparing the exclusion and inclusion conditions across Personal RateME and Cyberball revealed multiple neural activity differences between personal rejection and ostracism. Specifically, ostracism showed greater activity than personal rejection in bilateral areas, including the insula, the parietal and cingulate cortices, and the bilateral cerebellum. Conversely, we found greater activity in the bilateral hippocampus, precuneus, occipital and temporal cortices, and the paracentral gyrus during personal rejection compared with ostracism (Fig. 2). In terms of common activation related to exclusion during both tasks, we found that both personal rejection and ostracism were associated with reduced activity in parietal cortices and increased activity in the calcarine fissure (Fig. 2).

### Neural activity patterns of group rejection compared with personal rejection

**Fig. 3:**
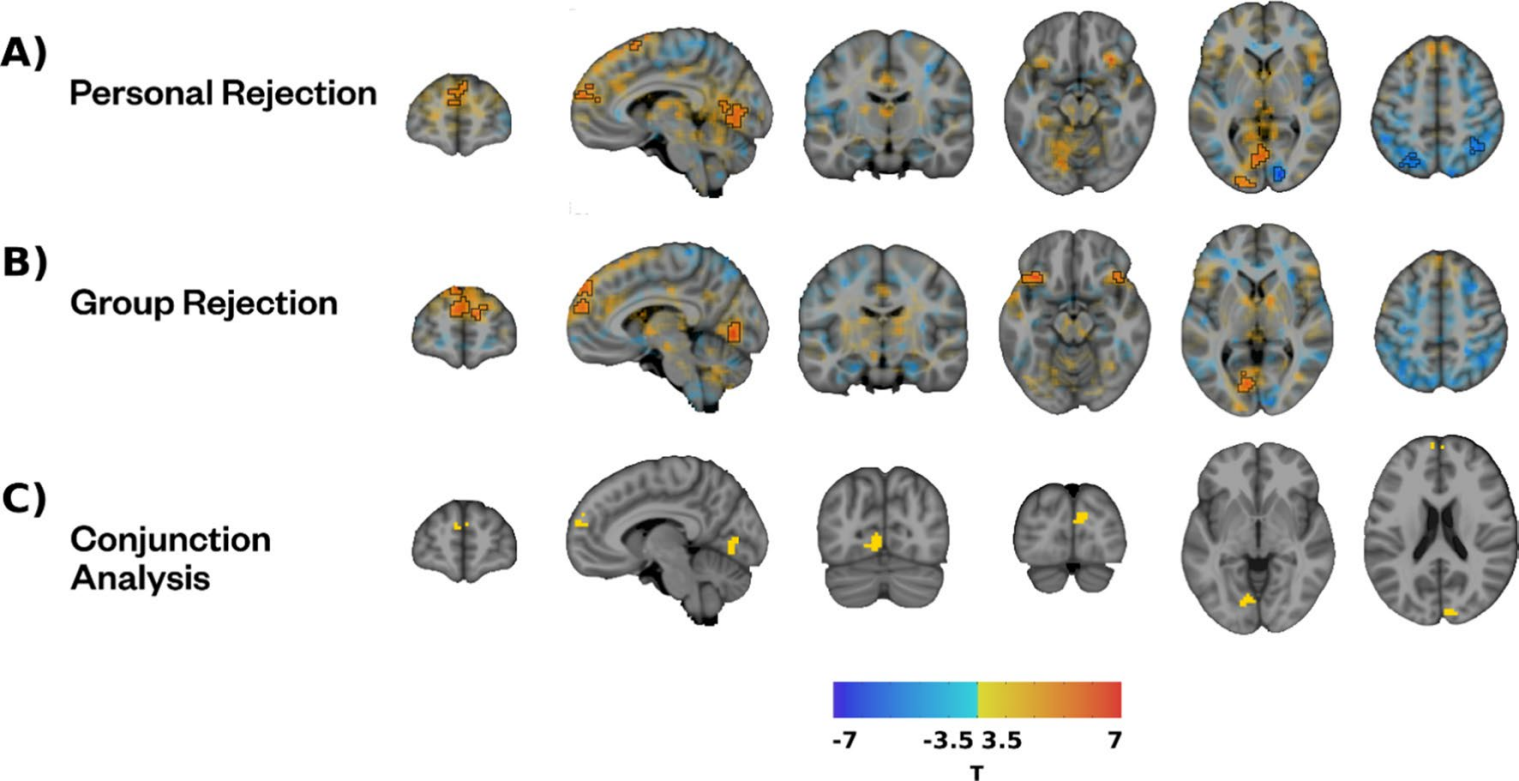
Activation maps characterizing rejection. *Note.* **A**) Activity related to personal rejection as the difference between exclusion and inclusion in Personal RateME. **B**) Activity related to group rejection as the difference between exclusion and inclusion during Group RateME. **C**) Results of the conjunction analysis between personal and group rejection

We found similar neural activity patterns in response to group rejection and personal rejection using group and personal RateME, respectively. Specifically, exclusion compared to inclusion during the group rejection task was associated with greater activity in the bilateral medial superior frontal gyrus, the ventral prefrontal cortex, and a cluster in the occipital lobe. We also found reduced activity in a cluster that is located mainly in the right cuneus in response to exclusion compared with inclusion (Fig. 3; Table 6). The comparison between personal and group RateME did not reveal any significant results. However, the conjunction analysis revealed common clusters of activity in the left lingual gyrus, the medial SFG, and the calcarine fissure, which presented greater activation during the exclusion compared with the inclusion condition during both tasks. Conversely, the right cuneus exhibited lower levels of activity during exclusion than.n both personal and group RateME.

**Table 6:** Neuroimaging results of the contrast between exclusion and inclusion in the Group RateME task

Cluster	Size	T mean	Overlap	ROI location
Exclusion > Inclusion				
1	136	4.01	64.40%	Left Superior frontal gyrus, medial
			26.80%	Right Superior frontal gyrus, medial
2	95	4.59	86.90%	Left Lingual gyrus
			10.80%	Left Calcarine fissure and surrounding cortex
3	41	4.03	53.50%	Right Inferior frontal gyrus, orbital part
			41.70%	Right Posterior orbital gyrus
4	38	4.32	58.90%	Left Posterior orbital gyrus
			32.10%	Left Inferior frontal gyrus, orbital part
Inclusion > Exclusion				
5	40	−4.26	81.00%	Right Cuneus
			32.10%	Left Cuneus

### Modulation of the neural response to group rejection by identity fusion and intergroup attitudes

**Fig. 4:**
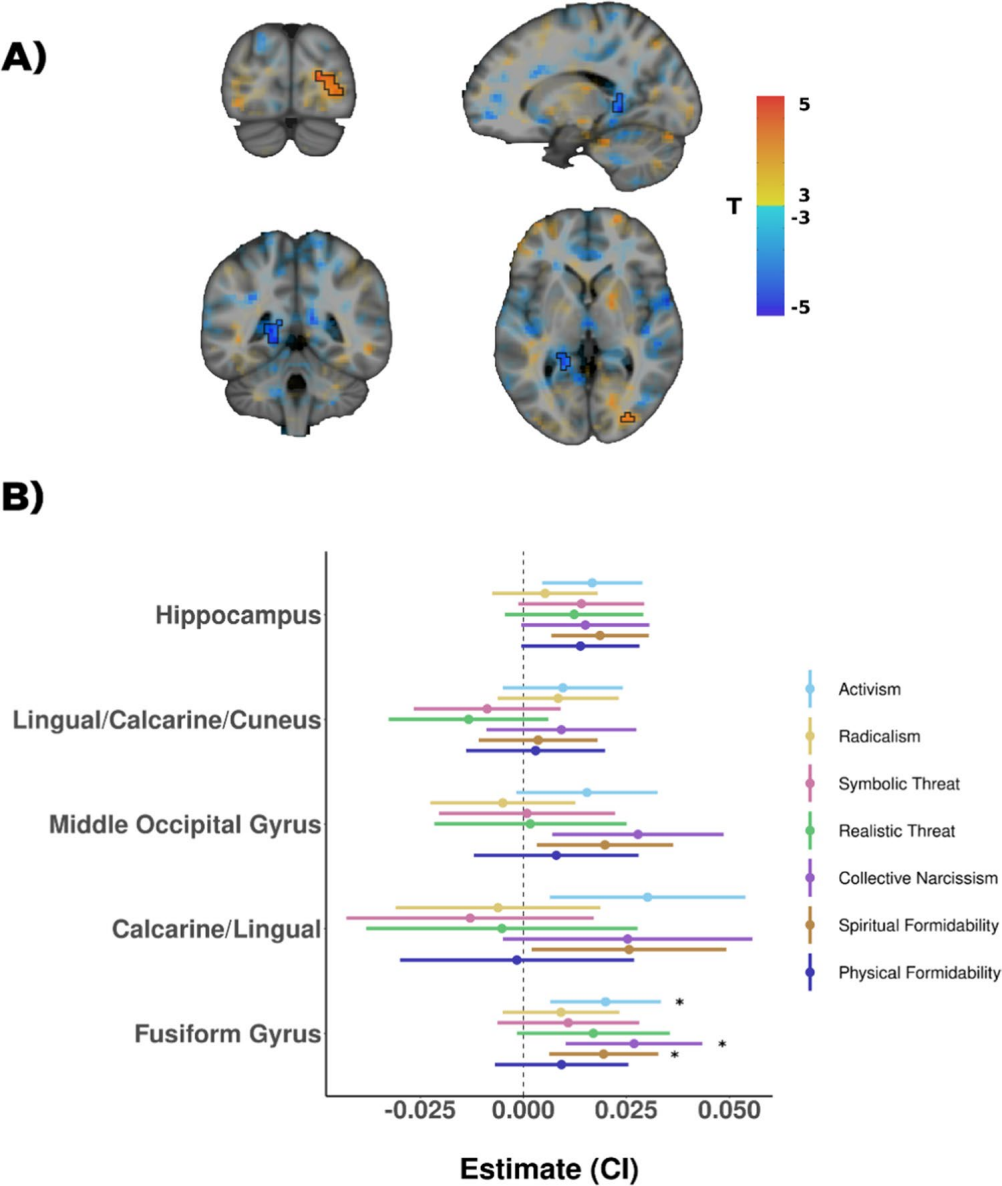
Analysis of identity fusion and intergroup attitude scales. *Note.* **A**) Maps of the modulatory effect of identity fusion on the activity related to group rejection (exclusion *versus* inclusion) at a threshold of *p* < 0.005 and 24 contiguous voxels. **B**) ROI analysis of the correlation between intergroup attitudes and group rejection

We examined whether identity fusion with the rejected group modulated neural responses to group rejection. These analyses did not yield significant results at p_FWE < 0.05. However, we depict the results with a more lenient threshold of *p* < 0.005 and the same cluster extent of 24 voxels for exploratory purposes (Fig. 4). We used this mask to extract the overlapping regions of a functional parcellation as described in the *Methods* section. Then, we correlated the activity levels of these regions during group rejection with the intergroup attitude scales. These analyses showed that the activity of the bilateral fusiform gyrus during group rejection was modulated by three intergroup attitude scales, including Activism (*b* = 0.020, *SE* = 0.008, 95% CI 0.006–0.033, *t* (50) = 2.435, *p* = 0.019, *q* = 0.045)*,* Ingroup Spiritual Formidability (*b* = 0.019, *SE* = 0.008, 95% CI 0.006–0.033, *t* (50) = 2.419, *p* = 0.019, *q* = 0.045), and Collective Narcissism (*b* = 0.027, *SE* = 0.010, 95% CI 0.010–0.043, *t*(50) = 2.663, *p* = 0.010, *q* = 0.045; Fig. 4). Detailed statistics of these analyses can be found in Supplementary Table 1.

## Discussion

We assessed neural activity patterns in response to group rejection. For that, we first characterized the neural correlates of social rejection compared with ostracism and then compared group-level and personal-level rejection. We used a novel social rejection task, “RateME,” to elicit feelings of group and personal rejection as well as Cyberball to induce feelings of ostracism during an fMRI acquisition. As predicted, we found neural activity patterns associated with social rejection to be different from ostracism. Particularly, social rejection recruited brain regions associated with autobiographical memory and self-identity, while ostracism activated regions related to social pain and salience. Furthermore, group-level and personal-level rejection exhibited similar neural activity patterns independently of how much participants identified with the groups being rejected against. Thus, group membership seems enough for group rejection to activate self-referential processing pathways similar to those activated by personal rejection.

Social rejection showed substantial neural activity differences relative to ostracism. We focused on personal rather than group rejection compared ostracism, because both Personal RateME and Cyberball took place in an interpersonal setting. Personal rejection showed greater activation in the Lingual Gyrus, the Supplementary Motor Area (SMA), and a part of the medial Superior Frontal Gyrus known as the Dorsomedial Prefrontal Cortex (DMPFC). These results partly align with a previous study that reports DMPFC and SMA activation in response to personal rejection using a task where participants viewed photographs of ex-partners and ex-friends (Woo et al., 2014). While the DMPFC is activated when inferring other people’s mental states (Isoda & Noritake, 2013), and when representing the self (Levorsen et al., 2023), the SMA has been involved in the regulation of negative emotions (Wager et al., 2008). Likewise, the Lingual Gyrus has also been found to be active during social rejection (Premkumar, 2012), as it responds to facial emotional expressions and self-representation (Levorsen et al., 2023; Palejwala et al., 2021). Conversely, ostracism triggered neural activity in brain regions associated with salience, attention, and disgust, including the Anterior Cingulate Cortex and the right Insula (Chapman & Anderson, 2012; Gan et al., 2022; Menon & Uddin, 2010; Merritt et al., 2021; Rolls, 2019; von Düring et al., 2019), as well as the Ventromedial Prefrontal Cortex, involved in emotional regulation (Hänsel & von Känel, 2008) and subjective value computations (Shapiro & Grafton, 2020). These results suggest that emotional and attentional processes are more important for ostracism, while self-referential processes may be more relevant for personal rejection. Individuals may more easily connect experiences of rejection to their own identity, possibly updating their self-concept.

When directly comparing personal rejection and ostracism, we found ostracism to elicit greater activation mainly in brain areas related to salience and attention, such as the Insula and Supramarginal Gyrus (Merritt et al., 2021; von Düring et al., 2019). Conversely, personal rejection showed greater activity in brain regions associated with self-referential processes, including the Superior and Middle Temporal Gyrus (Sajonz et al., 2010), Lingual Gyrus (Wagner et al., 2013), Precuneus (Cabanis et al., 2013), and Hippocampus (Kurczek et al., 2015). Regarding shared neural activity, the conjunction analysis revealed that personal rejection and ostracism both exhibit decreased activation in the Parietal Cortex, a region involved in executive functions (Katsuki & Constantinidis, 2012; Osada et al., 2019). These results are in line with studies showing that social exclusion decreases cognitive control abilities, such as response inhibition (Wang & Sha, 2018), therefore increasing aggressive behavior (Lasko et al., 2022; Quarmley et al., 2022). Moreover, both ostracism and personal rejection elicited activation in a small region of the Calcarine Fissure attached to the Posterior Cingulate Cortex, an area associated with self-referential processes that was found to be reliably activated during ostracism in a previous meta-analysis (Mwilambwe-Tshilobo & Spreng, 2021). Overall, although personal rejection and ostracism have some regions in common, these results reinforce the idea that being ignored in a group setting activates attentional processes, while receiving negative attention triggers self-referential processes.

When comparing group-level and personal-level rejection, we did not find any significant neural activity differences. Against expectations, personal and group rejection were neurally indistinguishable across the whole sample, regardless of participants’ identity fusion with the rejected group. These results are in line with a previous study that found similar levels of psychological distress in response to both personal and group rejection regardless of identity fusion (Marcos-Vidal et al., 2024a, 2024b). These findings combined suggest that group membership may be enough for people to take group grievances personally. Furthermore, the conjunction analysis revealed common clusters of activity in the Lingual Gyrus, the DMPFC, and the Calcarine fissure, areas associated with self-referential processes (Levorsen et al., 2023), including in the context of social rejection (Mwilambwe-Tshilobo & Spreng, 2021; Premkumar, 2012; Woo et al., 2014). Of note, activity in the Ventrolateral Prefrontal and Orbitofrontal Cortices was significantly increased during group rejection and almost significant during personal rejection, and activity in the Parietal Cortex was significant during personal rejection and almost significant during group rejection. These close-to-significant results may be due to insufficient statistical power; therefore, future studies with larger samples may find common activity patterns in these areas.

Regarding intergroup attitudes, we found that participants with higher scores in Activism, Ingroup Spiritual Formidability, and Collective Narcissism exhibited higher neural activation in the Fusiform Gyrus during group rejection. The Fusiform Gyrus is thought to play a key role in face perception (Weiner & Zilles, 2016) and social categorization (Shkurko, 2013), especially in ingroup bias (Van Bavel et al., 2008). Therefore, our results suggest that individuals with higher perceived ingroup grandiosity and a stronger commitment towards their group may be more responsive to ingroup and outgroup distinctions when their group is rejected.

Our findings shed light on the relationship between perceived group rejection and violent extremism. Feelings of group humiliation seem to activate self-referential pathways in the brain similar to those activated by personal experiences of rejection. Importantly, group membership was enough to elicit these effects, highlighting the potential of group rejection to impact the neural and psychological processes of all members of a rejected group, regardless of their degree of identification. Given the devastating consequences of personal rejection on people’s psychological well-being and aggression, our results suggest that group rejection should be examined as a risk factor for mental health and violent extremism, especially among vulnerable populations that are at risk of joining extremist groups.

The present study is bound to several limitations. To evaluate the neural activation patterns of personal and group rejection, we used 30-s-long experimental blocks, which exceeds the optimal time for estimating the hemodynamic response (~ 15 s; Maus et al., 2010). However, the employed block duration is similar, and even shorter, than in other Cyberball studies (Chester et al., 2018; Nishiyama et al., 2015; Onoda et al., 2010). Another possible limitation is related to the lack of significant differences between fused and nonfused individuals, suggesting that group belongingness alone may elicit similar brain responses to personal and group rejection. However, our experimental design does not allow us to examine the effects of group belongingness, because most of our participants may have felt a sense of belonging with their country of origin. To observe individual differences related to group belongingness, future research could examine groups with which participants do not inherently feel connected, such as political parties or sports teams.

## Conclusions

We characterized the neural response to group rejection by comparing it to personal rejection and ostracism. We found that while ostracism triggered heightened activity in regions related to social pain and salience, both personal and group rejection predominantly activated areas linked to autobiographical memory and self-identity. Furthermore, we observed similar neural responses to personal and group rejection in individuals who belong to the rejected group regardless of their level of identification with it. These findings suggest that group members process group rejection in similar ways to personal rejection at a brain level, which could explain why group rejection elicits levels of aggression and psychological distress similar to personal rejection. These findings underscore the distinct yet overlapping mechanisms of the different types of social exclusion and highlight the importance of addressing group rejection to mitigate its adverse effects on people’s psychological well-being. This is particularly important for vulnerable populations at risk of joining extremist groups, and perhaps even at the level of whole communities and nations whose peoples perceive themselves to be marginalized.

## Supplementary information

Below is the link to the electronic supplementary material.

Supplementary file1 (DOCX 459 KB)

## Data Availability

The study materials are available at https://osf.io/pc4fs/, and the dataset is publicly available at https://openneuro.org/datasets/ds005375.

## Code availability

The code employed in the analyses is available at https://osf.io/pc4fs/ or https://github.com/SBL-Barcelona/RateME-fMRI. A standalone application of the RateME task is available upon request. 

## Declarations

### Open science statement

The study materials and code employed in the analyses are available at https://osf.io/pc4fs/, and the dataset is publicly available at https://openneuro.org/datasets/ds005375. The hypotheses and analysis plan of this study were not preregistered.
