# Supplementary material for: When group grievances become personal: The neural correlates of group and personal rejection
Source: Cogn Affect Behav Neurosci. 2025 Jan 7;25(3):799–813. doi: 10.3758/s13415-024-01257-x (PMC12130114; doi:10.3758/s13415-024-01257-x)

**Supplementary Materials**

**Supplementary Methods**

**Protocol**

All participants completed an initial survey on demographic information and group identification measures. Next, participants were asked to complete a pre-scan survey in preparation for the intra-scanner RateME tasks. In the pre-scan survey, participants were asked to rate other people's profiles based on their name, age, hobbies, music preferences, and preferred vacation plans (see Table 1). This information was collected as part of the cover story for the Personal RateME task. Then, they were asked to rate different aspects of various European countries and their inhabitants (see Table 1). This information was collected as part of the cover story for the Group RateME task. All ratings were answered using a 5-star scale.

Before the neuroimaging session, participants completed a short training session, where they became familiar with the RateME tasks in the inclusion condition. Next, participants were invited to the fMRI facilities where they completed the three experimental tasks described below (Cyberball, Personal RateME, and Group RateME). Each participant underwent both of the conditions (inclusion and exclusion) in each experimental task.

**fMRI acquisition and preprocessing**

MRI scanning was performed in a Philips Ingenia 3.0T CX at the Fundació Pasqual Maragall in Barcelona. We collected a T1-weighted structural image before the functional images with an Ultra fast gradient echo sequence (echo time: 3.56ms, repetition time: 8ms, flip angle: 8º, matrix dimensions: 384 x 384 x 180, voxel size: 0.65 x 0.65 x 1 mm). After that, we acquired fMRI images using an echo-planar imaging (EPI)-T2* sequence (echo time: 35ms, repetition time: 1.75s, flip angle: 70º, matrix dimensions: 80 x 80 x 48, voxel size: 3 x 3 x 3 mm) that includes 261 volumes for each task.

Image preprocessing was performed using FMRIPREP version 20.2.0 [RRID:SCR_016216], a Nipype [RRID:SCR_002502] based tool. Each T1w (T1-weighted) volume was corrected for INU (intensity non-uniformity) using N4BiasFieldCorrection v2.1.0 and skull-stripped using antsBrainExtraction.sh v2.1.0 (using the OASIS template). Spatial normalization to the ICBM 152 Nonlinear Asymmetrical template version 2009c [ RRID:SCR_008796] was performed through nonlinear registration with the antsRegistration tool of ANTs v2.1.0 [RRID:SCR_004757], using brain-extracted versions of both T1w volume and template. Brain tissue segmentation of cerebrospinal fluid (CSF), white-matter (WM) and gray-matter (GM) was performed on the brain-extracted T1w using fast (FSL v5.0.9, RRID:SCR_002823).

Functional data was motion corrected using mcflirt (FSL v5.0.9). This was followed by co-registration to the corresponding T1w using boundary-based registration with six degrees of freedom, using flirt (FSL). Motion correcting transformations, BOLD-to-T1w transformation and T1w-to-template (MNI) warp were concatenated and applied in a single step using antsApplyTransforms (ANTs v2.1.0) using Lanczos interpolation.

Physiological noise regressors were extracted applying CompCor. Principal components were estimated for the two CompCor variants: temporal (tCompCor) and anatomical (aCompCor). A mask to exclude signal with cortical origin was obtained by eroding the brain mask, ensuring it only contained subcortical structures. Six tCompCor components were then calculated including only the top 5% variable voxels within that subcortical mask. For aCompCor, six components were calculated within the intersection of the subcortical mask and the union of CSF and WM masks calculated in T1w space, after their projection to the native space of each functional run. Frame-wise displacement was calculated for each functional run using the implementation of Nipype.

Many internal operations of FMRIPREP use Nilearn [RRID:SCR_001362], principally within the BOLD-processing workflow. For more details of the pipeline see <https://fmriprep.readthedocs.io/en/20.2.0/workflows.html>.

**Table S1.** Neuroimaging results of the contrast between exclusion and inclusion in Cyberball.

| **Cluster** | **Size** | **T mean** | **Overlap** | **ROI Location** |
| --- | --- | --- | --- | --- |
| 1 | 2135 | -4.73 | 17.9% | Left Inferior parietal gyrus |
|  |  |  | 14.4% | Left Precuneus |
|  |  |  | 13.3% | Right Inferior parietal gyrus |
|  |  |  | 11.3% | Right Precuneus |
|  |  |  | 10.6% | Left Superior parietal gyrus |
| 2 | 697 | -4.63 | 50.0% | Right Superior frontal gyrus, medial |
|  |  |  | 23.6% | Right Middle frontal gyrus |
|  |  |  | 11.1% | Right Inferior frontal gyrus, opercular part |
| 3 | 600 | -5.39 | 30.7% | Left Precentral gyrus |
|  |  |  | 26.3% | Left Superior frontal gyrus, medial |
|  |  |  | 24.6% | Left Supplementary motor area |
| 4 | 541 | 4.33 | 35.9% | Right Rolandic operculum |
|  |  |  | 27.6% | Right Superior temporal gyrus |
|  |  |  | 13.8% | Right insula |
| 5 | 316 | 4.24 | 42.4% | Left Superior temporal gyrus |
|  |  |  | 29.1% | Left Rolandic operculum |
|  |  |  | 14.1% | Left Postcentral gyrus |
| 6 | 166 | -4.32 | 62.3% | Left Precentral gyrus |
|  |  |  | 13.1% | Left Inferior frontal gyrus, triangular part |
|  |  |  | 12.2% | Left Middle frontal gyrus |
|  |  |  | 11.5% | Left Inferior frontal gyrus, opercular part |
| 7 | 163 | 3.95 | 30.4% | Left Anterior cingulate cortex, supracallosal |
|  |  |  | 15.3% | Left Superior frontal gyrus, medial |
|  |  |  | 15.3% | Left Anterior cingulate cortex, pregenual |
| 8 | 159 | 4.33 | 31.1% | Left Inferior frontal gyrus, triangular part |
|  |  |  | 26.3% | Left Inferior frontal gyrus, orbital part |
|  |  |  | 11.0% | Left Lateral orbital gyrus |
|  |  |  | 10.0% | Left Temporal pole: superior temporal gyrus |
| 9 | 154 | 4.07 | 45.4% | Left Middle cingulate & paracingulate gyri |
|  |  |  | 31.6% | Right Middle cingulate & paracingulate gyri |
|  |  |  | 11.0% | Right Supplementary motor area |
| 10 | 137 | -4.32 | 43.2% | Left Insula |
|  |  |  | 41.9% | Left Lenticular nucleus, Putamen |
| 11 | 117 | -4.26 | 21.2% | Left Thalamus, Ventral lateral Nucleus |
|  |  |  | 19.0% | Left Thalamus, Ventral posterolateral Nucleus |
|  |  |  | 12.6% | Left Thalamus, Intralaminar Nucleus |
| 12 | 78 | -4.33 | 67.9% | Lobule VI of cerebellar right hemisphere |
|  |  |  | 21.0% | Crus I of cerebellar right hemisphere |
| 13 | 77 | -4.06 | 68.3% | Left Middle occipital gyrus |
|  |  |  | 30.0% | Left Middle temporal gyrus |
| 14 | 72 | 4.06 | 83.6% | Right Precentral gyrus |
|  |  |  | 10.3% | Right Postcentral gyrus |
| 15 | 63 | 3.96 | 25.7% | Right Anterior orbital gyrus |
| 16 | 63 | 4.35 | 72.5% | Right Precentral gyrus |
|  |  |  | 24.0% | Right Postcentral gyrus |
| 17 | 46 | 3.82 | 59.0% | Left Calcarine fissure and surrounding cortex |
|  |  |  | 26.9% | Left Precuneus |
|  |  |  | 13.4% | Left Lingual gyrus |
| 18 | 43 | -4.38 | 72.9% | Crus I of cerebellar left hemisphere |
|  |  |  | 25.0% | Lobule VI of cerebellar left hemisphere |
| 19 | 33 | 3.89 | 95.6% | Left Postcentral gyrus |
| 20 | 30 | 3.86 | 95.5% | Left Postcentral gyrus |
| 21 | 28 | 3.9 | 63.9% | Left Cuneus |
|  |  |  | 30.1% | Left Superior occipital gyrus |
| 22 | 28 | -3.91 | 36.3% | Right Thalamus, Ventral lateral Nucleus |
|  |  |  | 18.8% | Right Thalamus, Mediodorsal lateral parvocellular Nucleus |
|  |  |  | 13.8% | Right Thalamus, Intralaminar Nucleus |
| 23 | 26 | -3.91 | 63.6% | Crus I of cerebellar left hemisphere |
|  |  |  | 34.8% | Lobule VI of cerebellar left hemisphere |
| 24 | 24 | 4.09 | 73.6% | Right Insula |
|  |  |  | 12.5% | Right Inferior frontal gyrus, opercular part |

**Table S2.** ROI analysis results of the modulatory effect of intergroup attitudes on group discrimination.

| **ROI** | **Scale** | **b** | **SE** | **CI** | **T (50)** | **p** | **q** |
| --- | --- | --- | --- | --- | --- | --- | --- |
| *Hippocampus* |  |  |  |  |  |  |  |
|  | Activism | 0.017 | 0.007 | [0.005, 0.029] | 2.256 | 0.028 | 0.100 |
|  | Radicalism | 0.005 | 0.008 | [-0.008, 0.018] | 0.674 | 0.295 | 0.353 |
|  | Symbolic Threat | 0.014 | 0.009 | [-0.001, 0.029] | 1.519 | 0.303 | 0.353 |
|  | Realistic Threat | 0.012 | 0.010 | [-0.004, 0.029] | 1.207 | 0.138 | 0.242 |
|  | Collective Narcissism | 0.015 | 0.009 | [-0.001, 0.031] | 1.594 | 0.010 | 0.045 |
|  | Spiritual Formidability | 0.019 | 0.007 | [0.007, 0.030] | 2.593 | 0.012 | 0.087 |
|  | Physical Formidability | 0.014 | 0.009 | [-0.001, 0.028] | 0.938 | 0.118 | 0.189 |
| *Lingual/Calcarine/ Cuneus* |  |  |  |  |  |  |  |
|  | Activism | 0.010 | 0.009 | [-0.005, 0.024] | 1.084 | 0.284 | 0.590 |
|  | Radicalism | 0.008 | 0.009 | [-0.006, 0.023] | 0.944 | 0.350 | 0.590 |
|  | Symbolic Threat | -0.009 | 0.011 | [-0.027, 0.009] | -0.811 | 0.421 | 0.590 |
|  | Realistic Threat | -0.013 | 0.012 | [-0.033, 0.006] | -1.130 | 0.264 | 0.590 |
|  | Collective Narcissism | 0.009 | 0.011 | [-0.009, 0.027] | 0.834 | 0.408 | 0.590 |
|  | Spiritual Formidability | 0.004 | 0.009 | [-0.011, 0.018] | 0.409 | 0.684 | 0.773 |
|  | Physical Formidability | 0.003 | 0.010 | [-0.014, 0.020] | 0.290 | 0.773 | 0.773 |
| *Middle Occipital Gyrus* |  |  |  |  |  |  |  |
|  | Activism | 0.015 | 0.010 | [-0.002, 0.033] | 1.482 | 0.145 | 0.337 |
|  | Radicalism | -0.005 | 0.011 | [-0.023, 0.013] | -0.467 | 0.643 | 0.900 |
|  | Symbolic Threat | 0.001 | 0.013 | [-0.020, 0.022] | 0.069 | 0.946 | 0.946 |
|  | Realistic Threat | 0.002 | 0.011 | [-0.022, 0.025] | 0.120 | 0.905 | 0.946 |
|  | Collective Narcissism | 0.028 | 0.010 | [0.007, 0.049] | 2.200 | 0.032 | 0.192 |
|  | Spiritual Formidability | 0.019 | 0.008 | [0.003, 0.036] | 1.966 | 0.055 | 0.192 |
|  | Physical Formidability | 0.008 | 0.010 | [-0.012, 0.028] | 0.656 | 0.515 | 0.900 |
| *Calcarine/Lingual* |  |  |  |  |  |  |  |
|  | Activism | 0.030 | 0.014 | [0.006, 0.0354 | 2.090 | 0.042 | 0.280 |
|  | Radicalism | -0.006 | 0.015 | [-0.031, 0.019] | -0.407 | 0.686 | 0.928 |
|  | Symbolic Threat | -0.013 | 0.018 | [-0.043, 0.017] | -0.708 | 0.482 | 0.844 |
|  | Realistic Threat | -0.005 | 0.020 | [-0.038, 0.028] | -0.260 | 0.796 | 0.928 |
|  | Collective Narcissism | 0.025 | 0.018 | [-0.005, 0.056] | 1.376 | 0.175 | 0.408 |
|  | Spiritual Formidability | 0.026 | 0.014 | [0.002, 0.049] | 1.788 | 0.080 | 0.280 |
|  | Physical Formidability | -0.002 | 0.017 | [-0.030, 0.027] | -0.091 | 0.928 | 0.928 |
| *Fusiform Gyrus* |  |  |  |  |  |  |  |
|  | Activism | 0.020 | 0.008 | [0.006, 0.033] | 2.435 | 0.019 | 0.045 |
|  | Radicalism | 0.009 | 0.009 | [-0.005, 0.023] | 1.059 | 0.295 | 0.353 |
|  | Symbolic Threat | 0.011 | 0.010 | [-0.006, 0.028] | 1.041 | 0.303 | 0.353 |
|  | Realistic Threat | 0.017 | 0.011 | [-0.002, 0.036] | 1.507 | 0.138 | 0.242 |
|  | Collective Narcissism | 0.027 | 0.010 | [0.010, 0.043] | 2.663 | 0.010 | 0.045 |
|  | Spiritual Formidability | 0.019 | 0.008 | [0.006, 0.033] | 2.419 | 0.019 | 0.045 |
|  | Physical Formidability | 0.009 | 0.010 | [-0.007, 0.025] | 0.938 | 0.353 | 0.353 |

**Figure S1.** Bar plot showing the effect of exclusion in Personal Rejection and Ostracism across different ROIs. Only ROIs where the effect of exclusion is significantly different across tasks (cyberball vs personal RateME) are displayed.


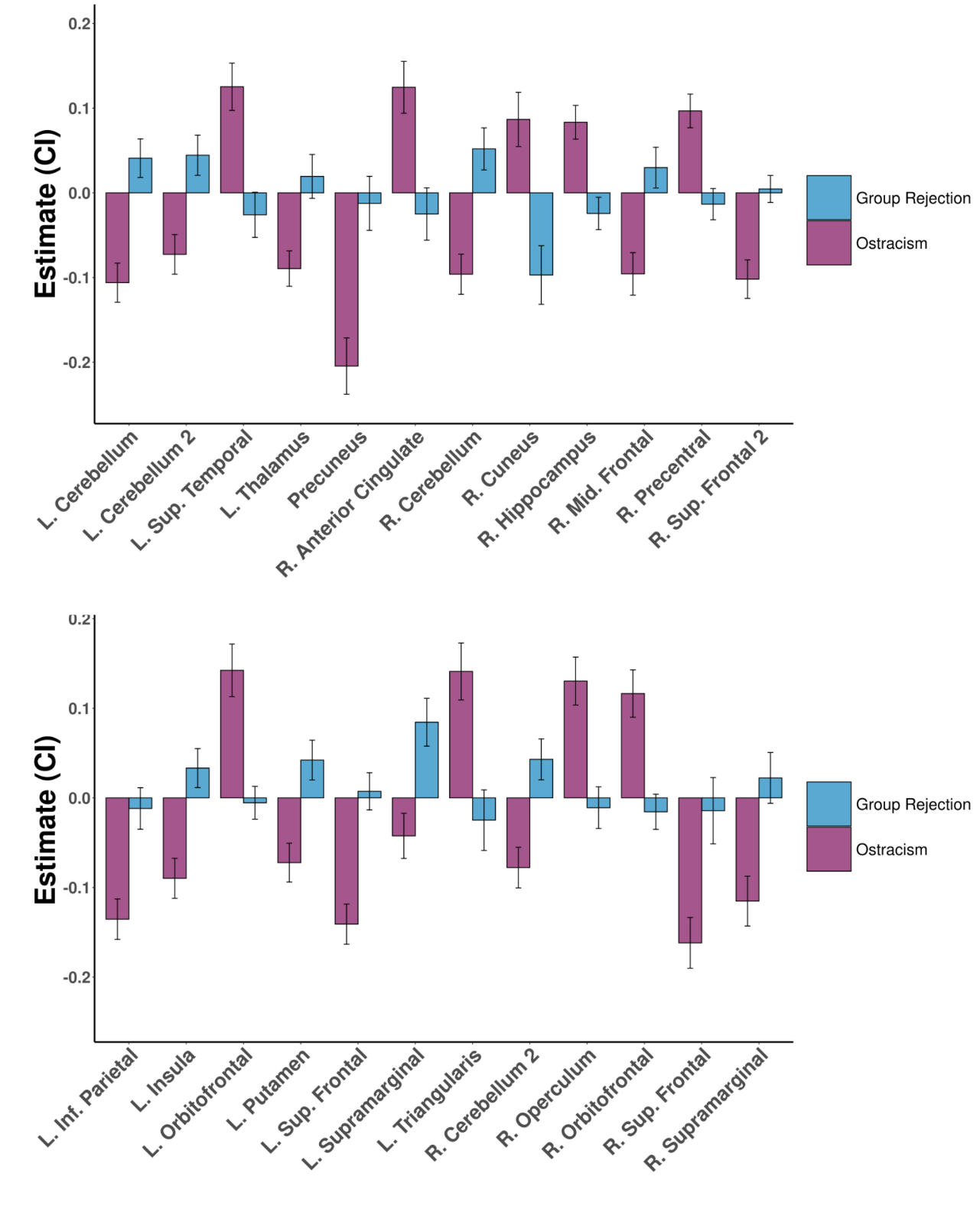


**Figure S2.** ROI analysis results of the modulatory effect of intergroup attitudes on group discrimination.


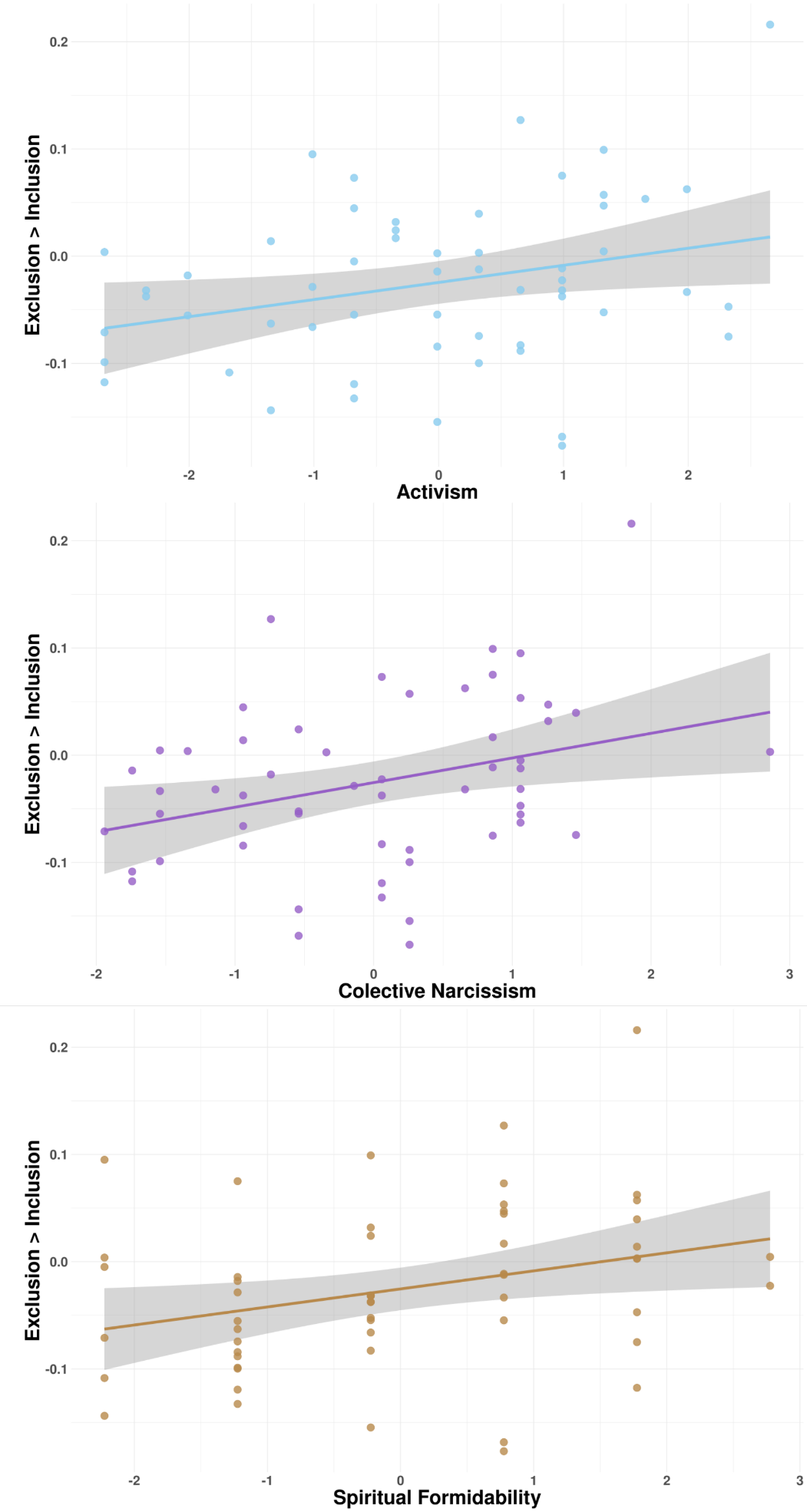

Supplement: Supplementary file 1 — Supplementary file1 (DOCX 459 KB) [file 13415_2024_1257_MOESM1_ESM.docx]
